# Time-trend analysis (2006 to 2019) of nutritional indicators among Brazilian pre-school children: making progress on child health inequalities

**DOI:** 10.1590/0102-311XEN122423

**Published:** 2023-10-23

**Authors:** Marly Augusto Cardoso

**Affiliations:** 1 Faculdade de Saúde Pública, Universidade de São Paulo, São Paulo, Brasil.

Nowadays, almost one out of three people suffer from at least one form of malnutrition in the world, including malnutrition, inadequate micronutrient status, overweight, obesity, and noncommunicable chronic nutrition-related diseases that affect social and economic development and health profile of individuals and populations. Since 1990, significant declines has occurred in global child mortality rates (52% in 2015). However, this figure is below the desired target (66% reduction) set out in the Millennium Development Goals agreed by the international community in 2000. A recent set of global targets have been agreed upon the Sustainable Development Goals (SDGs) to be achieved by 2030, including the SDG target 2.2 to end all forms of malnutrition among preeschool children ^1^.

The study by Castro et al. ^2^ examines the temporal trends of nutritional indicators in Brazilian preschool children by comparing the results of the *Brazilian National Survey on Demography and Health of Women and Children* (PNDS 2006) with those of the *Brazilian National Survey on Child Nutrition* (ENANI-2019) carried out in 2006/2007 and 2019, respectively. The authors also analyzed the time trend differences of these indicators using equiplot graph to depict regional inequalities (using the macroregions North, Northeast, Southeast, South, and Central-West), maternal schooling level (0-7, 8-10, 11 or ≥ 12 years of education) and self-reported maternal race/skin color (white, mixed-race, or black).

Reductions in regional, maternal education, and race/skin color disparities were observed for most nutritional indicators from 2006 to 2019. According to the ENANI-2019, the North Region of Brazil was the only exception, with childhood anemia increasing from 10.4% in 2006 to 17% in 2019. Although the prevalence of stunting reduced according to geographical regions and maternal education, it remained quite similar by maternal race/skin color from 2006 to 2019. In agreement with Castro et al. ^2^, these results can be related to the improvements in the living conditions and the expansion of public health and food and nutrition programs implemented from 2003 to 2015 ^3^. However, the recession and the public policies dismantling from 2016 onwards could explain the stability of the prevalence of stunting among children < 59 months of age over time, as changes in linear growth deficits may not be measured in immediate or intermediate periods ^4^.

In 2019, diet-related quality indicators among children 6-59 months of age suggested low dietary diversity with almost universal (88.8%) consumption of ultra-processed foods, which may explain the increasing trend in the prevalence of excessive weight from 2006 to 2019. Considering the relative stabilization of breastfeeding indicators in the same period and the persistent practice of abusive marketing of foods and ultra-processed foods, improvements in regulatory measures to protect children from the exposure to supply and marketing communication practices are urgently needed ^2^.

Castro et al. ^2^ also discuss the lack of full comparability between PNDS 2006 and ENANI-2019 regarding methodological differences in the measurement of anemia and vitamin A deficiency (since dried blood spots on filter paper used in PNDS 2006 has been considered reliable for vitamin A deficiency but not for anemia in preschool children ^5^). They also mentioned that the impossibility of taking prematurity into account in the PNDS 2006 could have overestimated the prevalence of stunting. However, the authors assumed that these limitations had negligible role when comparing the prevalence rates observed for these indicators in PNDS 2006 with ENANI-2019 results. On the other hand, different sampling procedures and size and management of missing data between the two surveys were not discussed. Possibly, such methodological differences could have contributed to underestimate the prevalence of anemia in the North Region in PNDS 2006 due to spatial heterogeneities of the participants, as previously suggested ^6^. The economic and social disparities among the North and other regions of Brazil require urgent implementation of nationwide public policies to minimize the impact of such inequalities on child nutrition.

Despite important progress in public health nutrition policies from 2003 to 2015 ^3^, children in rural areas tend to face more obstacles than their urban peers, including lack of access to quality healthcare and schools, among other factors. In addition to the data produced routinely by health information systems, population-based studies are needed to reflect the situation of those children, through direct collection of information on socioeconomic status, schooling, and ethnicity for monitoring health inequalities.

A number of challenges remain in monitoring nutritional indicators in Brazil. In addition to keep tracking the time series in urban and rural areas, one such challenge is improving the recruitment of individuals from socioeconomically deprived backgrounds who are more likely to be at risk for malnutrition - such as quilombola and indigenous children. The *First National Survey of Indigenous People’s Health and Nutrition in Brazil* was carried out in 2008/2009, including children < 5 years old and women from the macroregions of the country ^7^. Since then, no other national survey was performed. Among quilombola communities in the State of Alagoas (Northeast Brazil), for example, a major decline in prevalence of anemia in children 6-59 months of age was observed from 2008 (53%) to 2018 (38%), but anemia persisted as a relevant public health problem, especially in children 6-24 months of age (54%) ^8^. Racism, xenophobia, and discrimination are major determinants of child health, with potential intergenerational implications ^9^. Regular tracking of child nutritional status across different population groups is necessary to make progress towards increased equality and inclusivity ^9^.

I conclude this commentary by saluting all the researchers involved in the ENANI-2019, the Brazilian Ministry of Health that supported it, and my colleagues lead by Castro et al. ^2^ in this historical contribution to the understanding of child nutrition in Brazil just before the onset of the COVID-19 pandemic. The second edition of the ENANI is planned for 2024, allowing measuring the frequency of nutritional indicators for time series analysis using standardized sampling methodology, questionnaires, and measurement of biological parameters (anthropometry and collection of samples for laboratory tests).
